# A new section and species of AgaricussubgenusPseudochitonia from Thailand

**DOI:** 10.3897/mycokeys.40.26918

**Published:** 2018-09-20

**Authors:** Mao-Qiang He, Boontiya Chuankid, Kevin D. Hyde, Ratchadawan Cheewangkoon, Rui-Lin Zhao

**Affiliations:** 1 Department of Entomology and Plant Pathology, Faculty of Agriculture, Chiang Mai University, Chiang Mai, 50200, Thailand Institute of Microbiology, Chinese Academy of Sciences Beijing China; 2 State key laboratory of Mycology, Institute of Microbiology, Chinese Academy of Sciences, Beijing, 100101, China Chiang Mai University Chiang Mai Thailand; 3 Center of Excellence in Fungal Research, Mae Fah Luang University, Chiang Rai, 57100, Thailand Mae Fah Luang University Chiang Rai Thailand; 4 College of Life Sciences, University of Chinese Academy of Sciences, Huairou District, Beijing, 100408, China University of Chinese Academy of Sciences Beijing China

**Keywords:** New taxa, Agaricaceae, Phylogeny, Taxonomy

## Abstract

A large species diversity has recently been discovered in the genus *Agaricus*. Six subgenera and 23 sections are now recognised. In this study, three specimens collected from Thailand, formed a monophyletic clade in subgenus Pseudochitonia, based on analyses of ITS sequence data. Further analyses, based on multi-gene sequence data (ITS, LSU, tef1-α), using BEAST, revealed that this clade originated 26.7 Ma. According to their distinct morphological characteristics, phylogenetic position and relatively old divergence time, a new section Cymbiformes is proposed and this section is represented by a new species *A.angusticystidiatus*. This new section is characterised by the strong iodoform odour of basidiomes and cymbiform basidiospores. Descriptions, colour photographs and illustrations are presented.

## Introduction

*Agaricus* L. 1753 (Agaricaceae, Agaricales) is a well-known genus. Many species in this genus are commercially cultivated and served as food. One of the popular edible mushrooms is *A.bisporus* (J.E. Lange) Imbach, which is the most extensively cultivated mushroom in the world, accounting for 38% of world production (ISMS Edible mushrooms 2017, http://www.isms.biz/edible-mushrooms/). Another popular edible mushroom, *A.subrufescens* Peck, is also a medicinal mushroom and contains abundant bioactive compounds, for example, some compounds extracted from the basidiomes can be used as antioxidant (De Silva et al. 2012, 2013a, b, Llarena-Hernández et al. 2017). In the field, *Agaricus* is easily recognised by its white or brown caps with fibrillose scales on the surface, free lamellae, brown spore print and annulate stipe. Under the microscope, it is characterised by brown basidiospores, single or multiseptate cheilocystidia and often lacks pleurocystidia. Habitats of *Agaricus* are various, the most common being forests and grasslands, such as *A.campestris* L. of section Agaricus, which can be found gregariously in small groups or in fairy rings in grasslands. *Agaricus* also exists in arid habitats, for example, *A.colpeteorum* T. Lebel and *A.lamelliperditus* T. Lebel & M.D. Barrett of section Minores, which were discovered in arid zones of Australia (Lebel 2013).

The taxonomic, systematic and species delimitation of *Agaricus* inferred by morphology are variable (Cappelli 1984, Singer 1986). In the 1990s, the application of molecular techniques brought new perspectives to fungal taxonomic research including the genus *Agaricus* (White et al. 1990). Using phylogenetic analyses, the taxonomy of *Agaricus* is becoming more and more stable. Zhao et al. (2011) used ITS sequence data from *Agaricus* specimens from temperate and tropical areas to build a phylogenetic topology for the genus, which revealed eleven new clades and indicated phylogenetic relationships between temperate and tropical species. Zhao et al. (2016) carried out multi-gene phylogenetic and evolutionary molecular clock analyses. In that study, *Agaricus* was segregated into five subgenera and 20 sections, according to the phylogenetic position and divergence time of each clade. With the recent discovery of an American subgenus and a new clade found in the Caribbean area, *Agaricus* now contains six subgenera and 23 sections (Zhao et al. 2016, Chen et al. 2017, Parra et al. 2018).

In this study three interesting specimens found near Chiang Mai, Thailand were analysed morphologically and molecularly. We provide a full description and analyses are presented to support the distinction of this material as a new species and section in subgenus Pseudochitonia.

## Materials and methods

### Morphological examination

Photographs were taken immediately *in situ*, in the field in Thailand. Basidiomes were wrapped in aluminium foil or kept in plastic boxes separately. Macro morphological characteristics were recorded when specimens were fresh. Every specimen was completely dried in an electrical food drier at 60 °C, then kept in a plastic ziplock bag and deposited in Herbarium Mycologicum Academiae Sinicae (HMAS), Mae Fah Luang University Herbarium (MFLU), Biotec Bandkok Herbarium (BBH) and the Thiers Herbarium at San Francisco State University (SFSU). Colour terms and notations in parentheses are those of Kornerup and Wanscher (1978). Anatomical and cytological characteristics including basidiospores, basidia, cystidia and pileipellis were observed using an Olympus CX31 microscope. Scanning electron microscope (SEM) photos for basidiospores were captured through a Hitachi SU8010 Field Emission SEM (Tokyo, Japan). Measurements were analysed and recorded as X = the mean of length by width ± SD, Q = the quotient of basidiospore length to width and Q_m_ = the mean of Q values ± SD. All the protocols of morphological studies followed Largent’s methodology (Largent 1986).

### DNA extraction and PCR

At the Institute of Microbiology Chinese Academy of Science, genomic DNA was extracted from dry specimens by using an E.Z.N.A. Forensic DNA Extraction Kit (D3591-01, Omega Bio-Tek) following the manufacturer’s protocol. PCR amplification was performed following He et al. (2017). Primers for the internal transcribed spacer (ITS), large ribosomal subunit (LSU) and translation elongation factor (tef1-α) were ITS4/ITS5, LR5/LROR and 983f/1567r, respectively (White et al. 1990, Moncalvo et al. 2000, 2002, Morehouse et al. 2003). PCR products were sent to a commercial company for sequencing and both directions were sequenced to ensure accuracy. At the Botanic Garden Meise (BR), genomic DNA was extracted from dry specimens using a CTAB isolation procedure adapted from Doyle (1990). Ca. 10 mg of tissue was ground with a Retsch 300 beadmill. ß-mercaptoethanol (0.2%) was added to the CTAB lysis buffer just prior to extraction; samples were lysed for 1 hour at 60 °C; proteins and polysaccharides were removed by two consecutive extractions with chloroform: isoamylalcohol (24:1), after which DNA was precipitated by the addition of 0.8 volume isopropanol to the aqueous phase. The pellet was washed once in 600 μl 70% ethanol, air-dried and suspended in 100 μl TE pH 8.0. RNA was then digested with RNase A. For PCR amplification of the ITS1-5.8S-ITS2 region of rDNA, ITS1-F (Gardes and Bruns 1993) and ITS4 (White et al. 1990) primers were used. Amplifications were performed in 20 μl reactions containing 2 µl 10× polymerase buffer, 0.2 μM of each dNTP, 200 μg μl^-1^ bovine serum albumin (BSA), 0.25 μM of forward and reverse primers and 0.5 U Taq polymerase (DreamTaq, Thermo Scientific, St. Leon-Rot, Germany). Cycling was carried out using the following programme: 3 min at 94 °C; 35 cycles of 30 s at 94 °C, 30 s at 52 °C, 60 s at 72 °C; 5 min at 72 °C. PCR products were purified by adding 1 U of Exonuclease I and 0.5 U FastAP Alkaline Phosphatase (Thermo Scientific, St. Leon-Rot, Germany) and incubated at 37 °C for 1 h, followed by inactivation at 80 °C for 15 min. Sequencing was performed by Macrogen Inc. (The Netherlands) with PCR primers.

### Sequence alignment, phylogenetic analyses and divergence time estimation

A total of 119 specimens representing 87 species were incorporated in phylogenetic analyses. Three new sequences representing *A.angusticystidiatus* were generated from this study. They are one ITS sequence from specimen BC088 and two LSU sequences from ZRL2085 and ZRL2043 separately. Details of all sequences are listed in Table 1. Sequences were checked in BioEdit V.7.0.4 first (Hall 2007). Alignments were made by Muscle (Edgar 2004) for each region separately, then adjusted by hand and ambiguous regions removed. Alignments were submitted to TreeBase (Submission ID: 22231). Two data matrices were made for different analyses. The first one is an ITS sequence dataset which contains 84 specimens, all belonging to subgenus Pseudochitonia and an outgroup *A.campestris*. This dataset was used for Bayesian and Maximum Likelihood analyses. Phylogenetic trees generated by Bayesian Inference (BI) analysis were performed in MrBayes 3.1.2. (Ronquist and Huelsenbeck 2003). Best model is GTR + I + G which was indicated by MrModeltest 2.2 (Nylander 2004). Ten million generations were run for six Markov chains and sampled every 100^th^ generation resulting in 100,000 trees. Burn-in was determined in Tracer v1.6 with effective sample sizes (ESS) higher than 200 (http://tree.bio.ed.ac.uk/software/tracer). Remaining trees were used to calculate Bayesian posterior probabilities (PP). Maximum Likelihood (ML) analysis and bootstrap values calculation were performed in raxmlGUI 1.5b1 using GTRGAMMA model with 1000 replicates (Silvestro and Michalak 2012). The second dataset included 63 ITS, 61 LSU and 59 tef1-α gene sequences from specimens representing the six subgenera of *Agaricus*. The second multi-gene dataset was used for divergence time estimation. Model selections were performed in jModel Test v. 2 (Darriba et al. 2012) for each gene separately. An XML file was generated in BEAUTI v. 1.8. Priors were set according to the previous fossil-calibrated analysis of Zhao et al. (2016). An independent Monte Carlo Markov Chain of 50 million generations was run and log states every 5,000 generations by BEAST v1.8 (Drummond et al. 2012). The log file was checked in Tracer v. 1.6 (Rambaut et al. 2014) to ensure ESS (Effective Sample Sizes) value higher than 200. An ultrametric maximum-clade-credibility (MCC) tree was summarised using TreeAnnotator 1.8, discarding 10% of states as burn-in and annotating clades with ≥ 0.8 posterior probability.

**Table 1.** Taxa information used in the phylogenetic analyses, new taxa are in bold, “T” refers to type.

Species Name	Collection Number	LSU	ITS	tef1-α	Origin
* Agaricus abruptibulbus *	ZRL2012005	KT951460	KT951356	KT951626	Yunnan, China
*A.albosquamosus* T	LD2012192	KT951520	KT951394	KT951636	Thailand
*A.amoenus* T	ZRL2010072	KT951524	KT951348	KT951638	Yunnan, China
*** A. angusticystidiatus ***	**BC088**	–	**MG888054**	–	**Thailand**
*** A. angusticystidiatus ***	**ZRL2085**	**MG835413**	**KT951434**	–	**Thailand**
***A.angusticystidiatus* T**	**ZRL2043**	**MG835412**	**JF691553**	–	**Thailand**
* A. atrodiscus *	LD2012185	KT951473	KT284912	KT951653	Thailand
* A. benesii *	LAPAG283	–	JF797179	–	Burgos, Spain
* A. bernardiformis *	CA433	KT951467	KT951321	KT951577	–
* A. biannulatus *	LAPAG611	–	JF896229	–	Sardinia, Italy
* A. biberi *	LAPAG687	KR006614	KM657919	KR006642	Hungary
* A. bingensis *	ADK1992	–	KJ540954	–	Atakora, Benin
* A. bisporiticus *	LD2012111	KT951507	KJ575611	KT951650	Thailand
* A. bisporiticus *	MCR25	–	KJ575608	–	Pakistan
* A. bisporus *	LAPAG446	KR006611	KM657920	KR006640	Burgos, Spain
* A. bitorquis *	CA427	KT951491	KT951320	KT951646	
* A. bitorquis *	WZR2012827	KT951492	KM657916	KT951647	Xingjiang, China
* A. bohusii *	LAPAG562	KR006613	KM657928	KR006641	Madrid, Spain
* A. boisseletii *	CA123	–	DQ182531	–	–
* A. brunneopictus *	ADK2564	–	JF514518	–	Plateau Atlantique, Bénin
*A.brunneopileatus* T	ZRL2012115	KT951489	KT951404	KT951587	Yunnan, China
* A. brunneosquamulosus *	LD2012105	–	KJ540968	–	Thailand
* A. brunneosquamulosus *	ZRL4017	–	JF691549	–	Thailand
* A. caballeroi *	AH44503	–	KJ575605	–	Spain
* A. campestris *	LAPAG370	KR006607	KM657927	KR006636	Madrid, Spain
* A. campestroides *	LAPAF2	–	JF727842	–	Plateaux, Togo
*A.candidolutescens* T	LD2012129	KT951525	KT951335	KT951616	Thailand
A. cf. bernardi	CA383	KT951469	KT951319	KT951576	
A. cf. goossensiae	ADK2171	–	JF514517	–	Borgou, Benin
* A. chiangmaiensis *	NTS113	–	JF514531	–	Thailand
* A. comtulus *	LAPAG724	KT951448	KT951332	KT951593	Burgos, Spain
*A.crassisquamosus* T	ZRL2012607	KT951510	KT951376	KT951645	Tibet, China
* A. cupressicola *	LAPAG889	KT951465	KT951334	KT951649	Roma, Italy
* A. desjardinii *	WZR2012907	KT951474	KM657901	KT951644	Xinjiang, China
*A.dilutibrunneus* T	ZRL2012010	KT951512	KT951358	KT951569	Yunnan, China
* A. dolichopus *	ZRL2012715	KT951502	KT951382	KT951573	Tibet, China
* A. dolichopus *	ZRL2014120	–	KT951433	–	–
* A. duplocingulatus *	ZRL3064	–	KJ540966	–	Thailand
*A.erectosquamosus* T	LD2012165	KT951509	KT951338	KT951565	Thailand
* A. erythrosarx *	MURU6080	–	JF495068	–	–
* A. freirei *	CA186	–	DQ185553	–	–
* A. fuscofibrillosus *	WC913	–	AY484684	–	–
* A. fuscopunctatus *	LD2012115	–	KJ575612	–	Thailand
* A. fuscovelatus *	RWK2100	–	KJ577973	–	–
* A. gennadii *	CA339	–	KT951318	KT951575	–
*A.grandiomyces* T	ZRL2012611	KR006624	KM657879	KR006652	Tibet, China
* A. gratolens *	ZRL3093	KT951488	JF691548	–	Thailand
* A. haematinus *	ZRL2109	–	KT951435	–	Thailand
* A. haematinus *	ZRL2136	–	JF691552	–	Thailand
* A. hondensis *	RWK1938	–	DQ182513	–	USA
* A. huijsmanii *	LAPAG639	KT951444	KF447889	KT951571	Navarra, Spain
* A. kunmingensis *	ZRL2012015	KT951506	KT951361	KT951642	Yunnan, China
* A. kunmingensis *	ZRL2012007	–	KT951427	–	Yunnan, China
*A.lamellidistans* T	ZRL3099	–	JF691556	–	Thailand
* A. laskibarii *	LAPAG115	–	AY943975	–	Landes, France
*A.leucocarpus* T	LD2012159	KX083981	KU975101	KX198048	Thailand
*A.leucolepidotus* T	LD201214	KT951519	KT951336	KT951635	Thailand
*A.linzhiensis* T	ZRL2012618	KT951503	KT951378	KT951582	Tibet, China
* A. litoralis *	LAPAG420	KT951483	KT951327	KT951572	Burgos, Spain
* A. litoraloides *	ZRL2011249	KT951523	KT951353	KT951580	Yunnan, China
* A. magnivelaris *	F2389	–	JF727851	–	–
* A. martinicensis *	F2815	KX084032	JF727855	KX198038	MartiniqueFrance
* A. megacystidiatus *	LD2012179	–	KF305946	–	Thailand
* A. microvolvatulus *	LD201271	KT951508	KJ575614	KT951651	Thailand
* A. murinocephalus *	ZRL3044	–	JF691555	–	Thailand
* A. nevoi *	LAPAG257	KR006606	KM657922	KR006635	Burgos, Spain
* A. nevoi *	LAPAG535	–	KT951330	KT951574	Teruel, Spain
* A. nigrobrunnescens *	DEH632	–	JX308267	–	Hawaii, USA
*A.nigrogracilis* T	ZRL2012014	KR006621	KM657882	KR006647	Yunnan, China
* A. niveogranulatus *	LD201124	–	KJ540959	–	Thailand
* A. padanus *	WZR2012903	KR006616	KM657903	KR006644	Xingjiang, China
*A.pallidobrunneus* T	ZRL2012358	KT951471	KT951370	KT951566	Yunnan, China
* A. parvitigrinus *	CA158	–	AY899267	–	–
* A. pattersoniae *	RWK1415	–	AY943974	–	–
* A. phaeolepidotus *	CA217	–	DQ185552	–	–
* A. pilosporus *	LAPAG227	–	KT951425	–	Burgos, Spain
* A. pseudolangei *	ZRL3012	–	JF691551	–	Thailand
* A. rufoaurantiacus *	LAPAM15	KX671708	KT951313	KT951641	Dominican Republic
* A. silvaticus *	ALG07 213		KT951307	KT951567	Algonquin, ON, Canada
* A. sinodeliciosus *	WZR2012822	KT951518	KM657907	KT951648	Xingjiang, China
* A. sordidocarpus *	LD201237	–	KJ540946	–	Thailand
* A. subrufescens *	ZRL2012722	KT951451	KT951383	KT951632	Yunnan, China
* A. subsaharianus *	ADK4732	–	JF440300	–	Ouagadougou, Burkina Faso
* A. sylvaticus *	LAPAG382	KR006608	KM657929	KR006637	Burgos, Spain
* A. sylvaticus *	ZRL2012013	KT951500	KT951360	KT951570	Thailand
* A. sylvaticus *	ZRL2012568	KT951501	KT951371	KT951568	Tibet, China
* A. tibetensis *	ZRL2012585	KR006633	KM657895	KR006658	Tibet, China
* A. tollocanensis *	CA235	–	AY703913	–	–
* A. toluenolens *	CA911	–	KJ540947	–	–
*A.trisulphuratus* complex	LAPAF7	KR006605	KM657924	KR006634	Plateaux, Togo
*A.trisulphuratus* complex	Swk079	KT951472	KT951343	KT951561	Lanjak-Entimau, Malaysia
*A.trisulphuratus* complex	ZRL2014023	–	KT951428	–	China
*A.trisulphuratus* complex	ZRL2014024	–	KT951429	–	China
*A.trisulphuratus* complex	ZRL2014030	–	KT951432	–	China
*A.trisulphuratus* complex	ZRL2132	–	JF691558	–	Thailand
* A. tytthocarpus *	ZRLWXH3077	KR006618	KM657889	KR006645	Fujian, China
* A. variabilicolor *	ZRL4002	–	KT951438	–	Thailand
* A. variabilicolor *	ZRL4007	–	KT951439	–	Thailand
* A. variabilicolor *	ZRL4012	–	KT951440	–	Thailand
* A. variicystis *	LD201228	–	KT951426	–	Thailand
*A.variicystis* T	LD201234	KT951517	KT951339	KT951562	Thailand
* A. xanthodermulus *	CA160	–	AY899273	–	–
* A. xanthodermus *	CA15	–	AY899271	–	–
* A. xanthodermus *	LAPAG387	KR006609	KM657923	KR006638	Soria, Spain
* A. xanthosarcus *	Goossens5415	–	JF514523	–	–
*A.* sp.	CA486	–	JF797189	–	–
*A.* sp.	CA820	–	JF727861	–	–
*A.* sp.	LD2012162	KT951493	KT951337	KT951563	Thailand
*A.* sp.	NT020	–	JF797197	–	Thailand
*A.* sp.	Swk014	KT951482	KT951342	KT951654	Lanjak-Entimau, Malaysia
*A.* sp.	ZRL133	KT951505	KT951344	KT951656	Thailand
*A.* sp.	ZRL2010010	KT951511	KT951347	KT951639	Thailand
*A.* sp.	ZRL2010099	KT951479	KT951349	KT951564	Yunnan, China
*A.* sp.	ZRL2012267	KT951504	KT951368	KT951655	Yunnan, China
*A.* sp.	ZRL2012629	KR006627	KM657890	KR006656	Tibet, China
*A.* sp.	ZRLWXH3078	KT951464	KT951464	KT951643	Fujian, China
*A.* sp.	ZRLWXH3161	KT951526	KT951391	KT951615	Guangdong, China
*A.* sp.	ZRLWXH3140	–	KT951441	–	Guangdong, China
*Heinemannomyces* sp.	ZRL185	KT951527	KT951346	KT951657	Thailand

## Results

**Figure 1.**
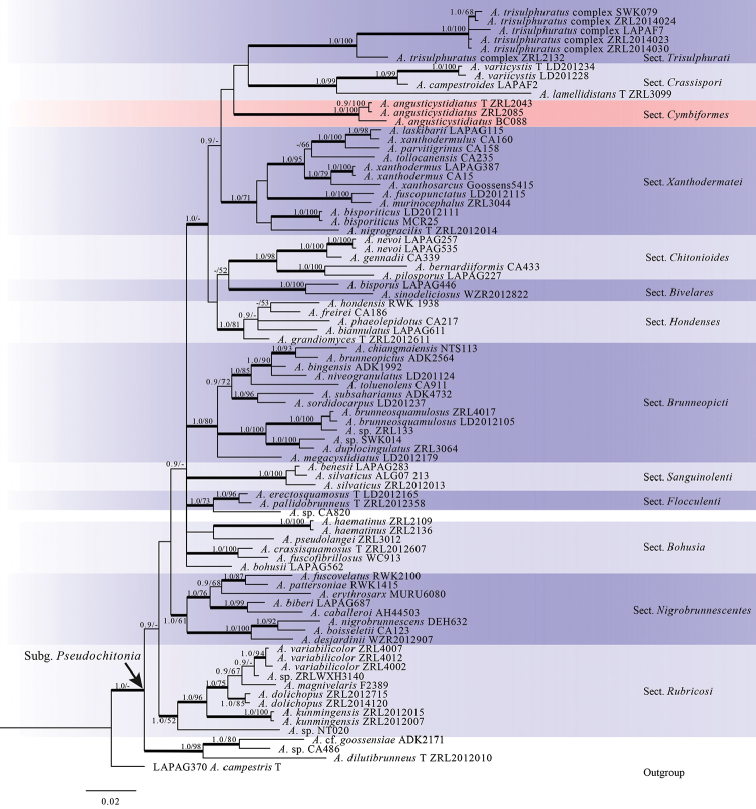
Phylogenetic tree of AgaricussubgenusPseudochitonia generated from Bayesian analysis of ITS sequences, rooted with *A.campestris*. Bayesian posterior probability (PP) values ≥ 0.9 or Bootstrap support (BS) values ≥ 50% are indicated at the internodes (PP/BS). The branches in bold mean the related PP > 0.95, “T” refers to sequences from type specimen.

The Bayesian tree from ITS sequences is shown in Figure 1. A total of 84 sequences are represented from 12 sections of subg. Pseudochitonia and *A.campestris* was used as outgroup. All sections are well supported both by posterior probabilities (PP) and bootstrap (BS). Phylogenetic trees generated from Bayesian and ML analyses showed identical topologies and are also almost identical with those of Zhao et al. (2016) with the exception of *A.dilutibrunneus* R.L. Zhao, which clustered with two unknown specimens (*A.* sp./CA486 and A.cf.goossensiae/ADK2171) and formed a monophyletic clade in our analyses, isolated from all other species in the previous study (Zhao et al. 2016). Our three specimens (*ZRL2043*, *ZRL2085* and *BC088*) formed a monophyletic clade in subg. Pseudochitonia which is fully supported both in PP and BS values and located at an isolated position (Fig. 1).

**Figure 2.**
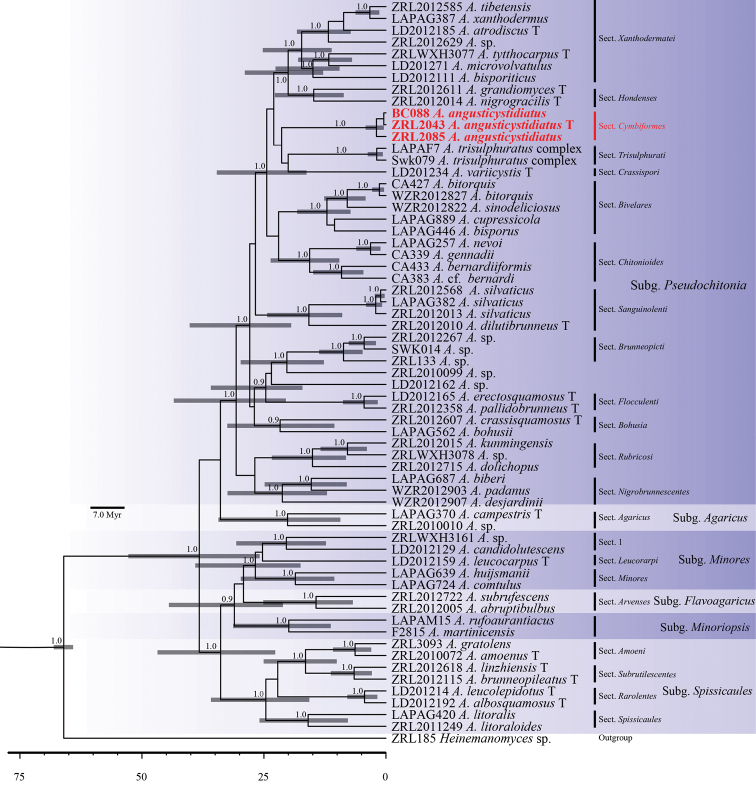
Maximium Clade Credibility tree of genus *Agaricus* based on ITS, LSU and tef1-α gene sequences with the outgroup *Heinemannomyces* sp. Posterior probability values equal or above 0.9 are annotated at the internodes. The 95% highest posterior density of divergence time estimation are marked by horizontal bars.

The multi-gene MCC tree is shown in Figure 2. It was conducted based on the dataset of multi-gene sequences. A total of 63 specimens were included, comprising 43 specimens used in ITS analysis, 19 specimens from five subgenera and an outgroup *Heinemannomyces* sp. All subgenera and sections are well-supported statistically. *Agaricus* diverged at the stem age 66 Ma (million years ago), all subgenera diverged between 29.2–33.9 Ma and sections diverged between 20–26.9 Ma. Our three specimens formed a new monophyletic clade in subg. Pseudochitonia with strong PP support and this clade diverged at 26.7 Ma.

### Taxonomy

#### Agaricus (Pseudochitonia) section Cymbiformes

Taxon classificationFungiAgaricalesAgaricaceae

M.Q. He & R.L. Zhao sect. nov.

MB824147

##### Type species.

*Agaricusangusticystidiatus* M.Q. He, Desjardin., K.D. Hyde & R.L. Zhao

##### Etymology.

In reference to the cymbiform basidiospores.

##### Original description.

KOH reaction negative, Schäffer’s reaction negative on dry specimens. No discolouration on touching, but discolouration reddish-brown on cutting. Annulus membranous. Smell strong iodoform. Basidiospores cymbiform and cheilocystidia narrow with variable shapes.

#### Agaricus angusticystidiatus

Taxon classificationFungiAgaricalesAgaricaceae

M.Q. He, Desjardin, K.D. Hyde & R.L. Zhao sp. nov.

MB825177

Figure 3

##### Etymology.

refers to the narrow clavate cheilocystidia.

##### Type.

Thailand, Chiang Mai Province, Mae Taeng, Baan Mae Sae village, on Hwy 1095 near 50 km marker, 19°14.599'N, 98°39.456'E, alt. 960 m. In rain forest dominated by *Castanopsisarmata*, *Castanopsis* sp., *Pinus* sp., *Lithocarpus* sp., 26 June 2005, collected by Jennifer Kerekes. **Holotype**: *ZRL2043* (HMAS279593); **Isotype**: BBH19428 and SFSUZRL2043,

##### Original description.

*Pileus* 40–80 mm diam., plano-convex, applanate, broadly umbonate; surface concentric squamulose with small skull-cup at disc, appressed, slightly fissured, light brown (6D8), brown (7E3), greyish-brown (5D5), dark brown (6D6) against the grey (8E3) background. Context 4–5 mm thick at disc, fragile, white to grey (8E3) in age. *Lamellae* free, crowded, lamellulae with 3–4 lengths, 3–4 mm broad, normal to slightly ventricose, brown (7E5) to dark brown (7F7-8), edge colour similar to the gill itself. *Stipe* 55–100 × 5–8 (base 8–15) mm, cylindrical bulbous, with rhizomorphs in most cases, hollow, surface glabrous to silky, white to dark brown (6D6). *Annulus* pendent or percurrent; single; upper side membranous, white; lower side surface powdery, light yellow (4B2) grain-like dots in circulate; superior, persistent, edge entire, up to 5 mm broad. Smell of iodoform. No colour change on touching; light dull red, greyish brown (7D4) on cutting.

KOH reaction: negative. Schäffer’s reaction: negative on dry specimens.

*Basidiospores* 5–6.5 × 3–4 (–4.5) µm [X = 5.6 ± 0.5 × 3.8 ± 0.4, Q = 1.1–2.2, Q_m_ = 1.52 ± 0.7, n = 20], cymbiform, some endosporium, no germ pore, brown. *Basidia* 10–15 × 5.5–7 µm, clavate, hyaline, smooth, 4-spored. *Pleurocystidia* absent. *Cheilocystidia* 20–30 (–45) × 5–8 µm, occasionally one septum, narrowly clavate to clavate, some with elongated top, rarely subcapitate, hyaline, smooth. *Pileipellis* cutis consisting of 3–5 µm diam. hyphae, hyaline, smooth, non-constricted at septa. Annulus hyphae same as pileipellis.

**Figure 3.**
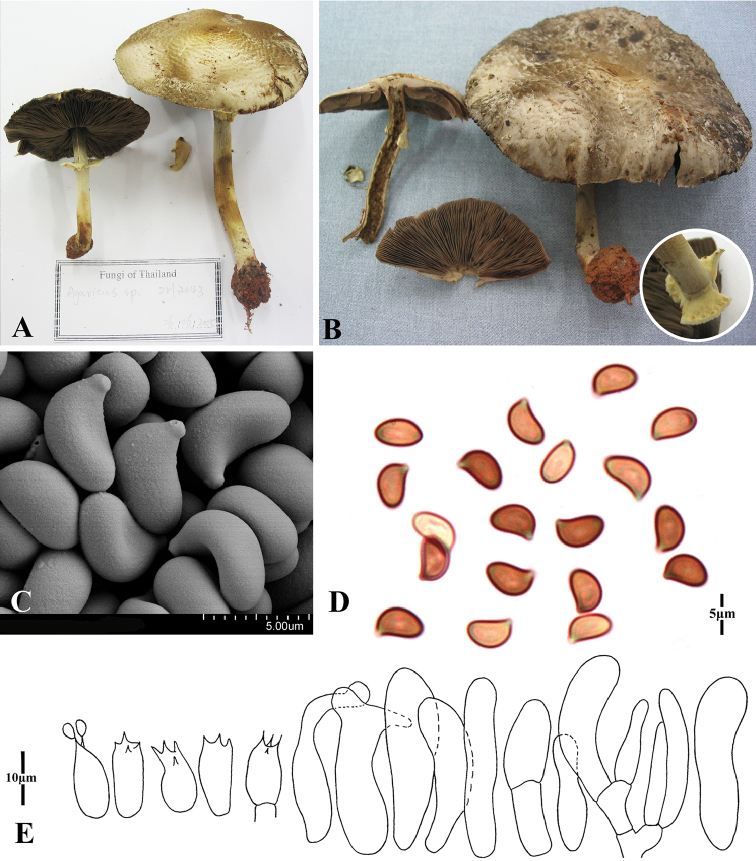
Morphology of *Agaricusangusticystidiatus***A, B** basidiomes **C, D** basidiospores **E** basidia and cheilocystidia.

##### Habit.

Gregarious on soil in rain forest which is mainly dominated by *Castanopsisarmata*, *Castanopsis* sp., *Pinus* sp., *Lithocarpus* sp.

##### Distribution.

Thailand, Chiang Mai Province (type distribution).

##### Other materials examined.

Thailand, Chiang Mai Province, Mae Taeng, Ban Mae Sae Village, on Hwy 1095 near 50 km marker, 19°14.599'N, 98°39.456'E, elev. ca. 960 m, 3 July 2004, collected by Thitiya Boonpratuang, *ZRL2085* (HMAS279594, BBH19468 and SFSUZRL2085); Thailand, Chiang Mai Province, Mae Taeng, Mushrooms research center, 30 July 2014, collected by Boontiya Chuankid, *BC088* (MFLU 14-0903).

##### Notes.

This new species is morphologically distinguished from other *Agaricus* species by its strong iodoform smell, context reddish-brown discolouration on cutting, cymbiform basidiospores and narrow cheilocystidia with variable shapes. Phylogenetic analyses confirmed it is a member of the subgenus Pseudochitonia with an isolated phylogenetic position in *Agaricus*. This new species is similar to *A.iodolens* Heinem. & Gooss.-Font. of section Xanthodermatei, because both have relatively slender basidiomes and odour of iodine (Naritsada et al. 2014). However, this new species has cymbiform basidiospores and a bulbous stipe, while those of *A.iodolens* are ellipsoid and an equal stipe (Zoberi 1972). *Agaricuslamellidistans* R.L. Zhao and *A.variicystis* L.J. Chen, K. D. Hyde & R. L. Zhao of section Crassispori resemble this new species, because all have greyish-brown pilei and cymbiform basidiospores. These species lack discolouration on cutting, while those of *A.angusticystidiatus* have dull red discolouration on cutting (Zhao et al. 2016).

## Discussion

Based on phylogenetic and morphological studies, we propose *A.angusticystidiatus* as a new species in subgenus Pseudochitonia. Furthermore, the dating analysis, based on multi-gene sequences, indicated that *A.angusticystidiatus* diverged at 26.7 Ma which is slightly older than other sections in *Agaricus* (18–26 Ma, in Zhao et al. 2016). Therefore, a new section Cymbiformes is proposed, which presently only contains species *A.angusticystidiatus*. Thus up to now, there are six subgenera and 24 sections in the genus *Agaricus* (Zhao et al. 2016; Chen et al. 2017; Parra et al. 2018).

Zhao et al. 2016 had conducted a reconstruction of the taxonomic system of *Agaricus*. In that study, they used the following criteria to recognise subgenera and sections: “(i) they must be monophyletic and statistically well-supported in the multi-gene analyses; (ii) their respective stem ages should be roughly equivalent and subgenera stem ages must be older than section stem ages; and (iii) they should be identifiable phenotypically, whenever possible” (Zhao et al. 2016). That means divergence time has been used as an additional criterion to rank taxa of above species level in *Agaricus*. Later, the criterion of divergence time, along with phylogenetic, monophyletic and morphological support, has been accepted in other new subgenus and section recognitions in *Agaricus*, such as a new subgenus Minoriopsis (Chen et al. 2017); and a new section Kerrigania (Parra et al. 2018).

As mentioned before, this proposed new section Cymbiformes has a closely phylogenetic relationship with sections *Trisulphurati* and *Crassispori*. In morphology, all of them differed with other sections of *Agaricus* by the combination of negative Schäffer’s reaction, chemical odours such as phenol, ink or carbolic acid and basidiospores endosporium and often cymbiform. However, section Trisulphurati has woolly squamules on the surfaces of the pileus and stipe and the other two sections only have appressed squamules at the centre of the pileus. Furthermore, this new section Cymbiformes could be separated from section Crassispori by its negative KOH reaction and developed annulus (the latter is positive KOH reaction and with fragile annulus) (Zhao et al. 2016).

So far, section Cymbiformes is only known from a tropical area. The cymbiform basidiospores are rare in *Agaricus* species. Presently there are three *Agaricus* species from tropical areas which have this kind of basidiospores. They are *A.angusticystidiatus* of section Cymbiformes and *A.lamellidistans* and *A.variicystis* of section Crassispori (Zhao et al. 2016). In phylogenetic analyses, these two sections also show a close phylogenetic position, which is similar to previous studies (specimens *ZRL2043* and *ZRL2085* were treated as *A.* sp. in Zhao et al. 2011; Zhao et al. 2016). The presence of cymbiform basidiospores is a common character in another genus *Micropsalliota* of Agaricaceae. In phylogenetic analyses, *Agaricus* is sister to *Hymenagaricus*, then sister to *Chlorophyllum, Heinemannomyces* and *Micropsalliota* (Zhao et al. 2017) and all of them have tropical distribution habitats. Thus we hypothesised that cymbiform basidiospores have formed at least twice in evolutionary events and are associated with tropical environments.

## Supplementary Material

XML Treatment for Agaricus (Pseudochitonia) section Cymbiformes

XML Treatment for Agaricus angusticystidiatus
